# Gender matters

**DOI:** 10.1192/j.eurpsy.2021.982

**Published:** 2021-08-13

**Authors:** P. García Vázquez, R. Gomez Martinez

**Affiliations:** Psiquiatría, Complejo Asistencial Universitario León, León, Spain

**Keywords:** bipolar disorder, seasonal pattern, Gender

## Abstract

**Introduction:**

Recently, the seasonal pattern of bipolar disorder has been accepted, with the clinical, diagnostic, treatment and prognostic consequences that this entails. It is interesting to study its epidemiological characteristics, such as the influence of gender on this pattern.

**Objectives:**

To study the influence of gender in the Seasonal Pattern of Bipolar Disorder.

**Methods:**

A systematic review was carried out by means of a bibliographic search in Ovid MEDLINE of articles published in the last ten years (2010-2020), using the following keywords: bipolar disorder, seasonal pattern and gender: Those studies carried out in patients who presented a seasonal pattern were selected, and the influence of gender on this was studied.

**Results:**

The initial search showed a total of 92 articles, of which 7 met the inclusion criteria. It was found that, indeed, gender influences both the clinical characteristics and the course, management and prognosis of the seasonality of bipolar disorder.

**Conclusions:**

The diagnosis of the Seasonal Pattern in Bipolar Disorder continues to be an important challenge. Women more frequently present PE, associated with manic, depressive or mixed episodes, while men in depressive episodes. Men are more frequently associated with Bipolar Disorder type II and depressive episodes, and women with rapid cycling and eating disorders. Male manic episodes are associated with psychotic symptoms, and with greater severity in admissions. Women have a higher risk of Seasonal Pattern than men, with the clinical and prognostic repercussions that this entails.

